# Optimizing active recovery strategies for finger flexor fatigue

**DOI:** 10.3389/fspor.2024.1480205

**Published:** 2024-12-12

**Authors:** Dominika Krupková, James J. Tufano, Jiří Baláš

**Affiliations:** Faculty of Physical Education and Sport, Charles University, Prague, Czech Republic

**Keywords:** rock climbing, near-infrared spectroscopy, oxygen saturation, sport climbing, intermittent exercise

## Abstract

**Introduction:**

Active recovery (AR) is used during exercise training; however, it is unclear whether the AR should involve the whole body, only the upper extremities, or only the lower extremities when aiming to maintain localized upper body performance. Therefore, this study aimed to evaluate the impact of different AR strategies on repeated intermittent finger flexor performance leading to exhaustion.

**Methods:**

A crossover trial involving a familiarization session and three laboratory visits, each including three exhaustive intermittent isometric tests at 60% of finger flexor maximal voluntary contraction separated by 22 min of randomly assigned AR: walking, intermittent hanging, and climbing.

**Results:**

The impulse (Nꞏs) significantly decreased from the first to third trials after walking (−18.4%, *P* = 0.002, *d* = 0.78), climbing (−29.5%, *P* < 0.001, *d* = 1.48), and hanging (−27.2%, *P* < 0.001, *d* = 1.22). In the third trial, the impulse from the intermittent test was significantly higher after walking (21,253 ± 5,650 Nꞏs) than after hanging (18,618 ± 5,174 Nꞏs, *P* = 0.013, *d* = 0.49) and after climbing (18,508 ± 4,435 Nꞏs, *P* = 0.009, *d* = 0.54).

**Conclusions:**

The results show that easy climbing or intermittent isolated forearm contractions should not be used as AR strategies to maintain subsequent performance in comparison to walking, indicating that using the same muscle group for AR should be avoided between exhaustive isometric contractions.

## Introduction

Repeated forearm isometric contractions are common in various daily work activities or sports such as canoe slalom or rock climbing, where limited working capacity can be a constraining factor for performance (1–3). During high-intensity localized contractions, increased intramuscular mechanical pressure leads to decreased blood flow and tissue oxygen saturation, inducing a greater reliance on fast glycolysis, factors that are connected to a drop in pH level and metabolite efflux (4, 5). Fatigue is, therefore, often associated with localized metabolic factors, which can result in a reduction in maximum force or power production (6, 7). However, to mitigate this reduction in performance during repeated bouts of localized high-intensity contractions, an appropriate recovery strategy may help (8).

Intra-session recovery strategies are generally passive or active. Active recovery (AR) is commonly used in sports involving repetitive performance. For instance, AR has been found to be beneficial between repetitive swimming or cycling sprints lasting 40–120 s with a recovery period exceeding 2 min (9, 10). However, various physiological and psychological variables may affect the final impact of AR on subsequent performance (11). Physiologically, AR facilitates metabolite removal, increases heart rate, and enhances blood flow to actively working muscles, possibly delaying the onset of subsequence fatigue (12–14). The degree of recovery depends on the type and degree of fatigue (11), AR intensity (15, 16), duration (17), and the form of AR (18, 19). However, the form of AR in terms of muscle group involvement has not been well explored.

To elaborate on the importance of the specific muscle groups involved in AR, some have speculated that AR involving different muscle groups is more beneficial than involving the same muscle groups between exercises (19, 20). For instance, both low-intensity leg AR and arm AR were more effective than passive recovery in extending pedaling time on a cycle ergometer, with arm AR proving less effective than leg AR in sustaining cycle ergometer performance, suggesting that involving the same muscle groups for AR may provide some benefits (19–21). However, conflicting conclusions exist regarding AR form between exhaustive forearm contractions. For example, Baker et al. (22) recommended leg AR between paddling sets for slalom canoeists, while Valenzuela et al. (18) found easy climbing to be a more beneficial recovery strategy than walking between two climbing performances.

While research has shown a superior effect of AR over passive recovery for exhaustive forearm exercise (12, 13, 23), it is unclear whether engaging the same muscle groups during AR involved in forearm performance may facilitate recovery. Engaging the same muscle groups in AR may increase localized blood flow and facilitate metabolic removal (24), but it may not allow for sufficient restoration of glycogen stores required for subsequent performance (25, 26). Furthermore, it is unknown whether AR should involve the whole body, only the upper extremities, or only the lower extremities when aiming to maintain localized upper body performance.

Therefore, this study aimed to evaluate the impact of different active recovery strategies on repeated finger flexor intermittent performance leading to exhaustion.

## Material and methods

### Experimental plan

Sport emphasizing upper limb strength and endurance mostly analyzed in the existing recovery literature has been sport climbing (12, 13, 18, 23). Very few other studies primarily focusing on upper limb muscles have examined the effect of AR on performance (27, 28). Due to the multifaced structure of sport climbing performance, we opted for an intermittent isolated test primarily involving the finger flexors, providing greater control over the physiological response. This test demonstrated high reliability (ICC = 0.907), with limits of agreement at 4 558.5 N.s (29) and criterion validity to climbing ability (30, 31).

**Figure 1:**
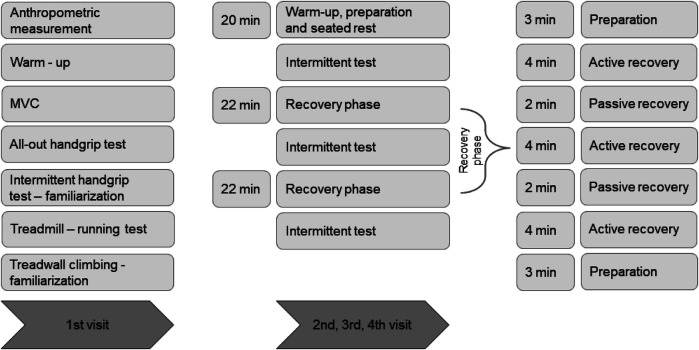
Design of the study indicating four visits of all participants. Maximal voluntary handgrip contraction (MVC). Active recovery—one randomly assigned active recovery for one session (climbing, walking, or hanging). Passive recovery—participants stayed calm in a sitting position.

The participants visited the laboratory four times separated by 3–6 days. During their first visit, they completed questionnaires concerning their ability level, climbing preferences, and experience and undertook anthropometric measurements (body mass, height). After a standardized warm-up (5 min of stair walking, 5 min mobilizing exercises, 5 min traversing on the climbing wall, and 5 min short bouts of submaximal intermittent hanging on various wooden rungs to activate forearm flexors), the participants completed the test of finger flexor maximal voluntary contraction (MVC), 4 min finger flexor all-out test (32), and familiarized themselves with the intermittent handgrip test at 60% MVC (29). An exhaustive treadmill running test with progressive increases in inclination (31) was used to determine heart rate (HR) max. At the end of the first visit, the participants familiarized themselves with a motorized climbing ergometer (tread wall). During each of the following visits, the participants performed, after standardized warm-up, three exhaustive intermittent isometric tests at 60% MVC with randomly assigned AR in between (1) walking; (2) isolated forearm contractions on a wooden rung—hanging; and (3) climbing. The experimental plan is depicted in Figure 1.

### Participants

Seventeen male sport climbers (age, 31.2 ± 8.6 years; body mass, 72.3 ± 7.9 kg; height, 176.9 ± 6.1 cm; mean ± SD) volunteered to participate in this study. They self-reported their climbing ability from 10 to 25 on the International Rock Climbing Research Association (IRCRA) scale, classifying themselves as intermediate, advanced, and elite athletes (33). The participants were healthy with a minimum of 3 years of climbing experience. Training characteristics from the last 3 months before the study are shown in Table 1. The participants were required to refrain from strenuous physical activity 24 h before testing and caffeine 12 h before testing. The study was approved by the institutional ethics committee in accordance with the Declaration of Helsinki. All participants were informed of the risks of the experiment and signed an informed consent. Sample size calculations were determined using G*Power (version 3.1.9.7) based on the outcome measures from previous research (34). With power (*β* error) set at 0.95, *α* level set at 0.05, and an effect size (eta squared) of 0.25 for differences in performance after different recovery strategies, the sample size was set as 15 participants. Because of possible dropout and technical errors, 17 participants were recruited.

**Table 1:** Training characteristics from the last 3 months (mean ± SD) and their association (*R*—Pearson correlation coefficient) with a drop in performance between the first and third test after hanging, walking, and climbing recovery.

		Δ Impulse	Δ Impulse	Δ Impulse
		Hanging (N.s)	Walking (N.s)	Climbing (N.s)
	Mean ± SD	6,969 ± 3,416	4,782 ± 4,658	7,743 ± 4,363
Climbing ability (IRCRA scale)	17 ± 4	*R* = 0.14	*R* = −0.01	*R* = −0.13
Climbing experience (years)	9.5 ± 6.9	*R* = −0.25	*R* = 0.12	*R* = 0.02
Climbing-specific training (hours/week)	4.2 ± 2.3	*R* = −0.16	*R* = −0.27	*R* = −0.47
Climbing non-specific training (hours/week)	3.4 ± 2.6	*R* = −0.28	*R* = −0.20	*R* = 0.45
Lead climbing (meters/week)	199 ± 124	*R* = −0.17	*R* = 0.17	*R* = −0.05
Bouldering (movements/week)	151 ± 140	*R* = 0.20	*R* = 0.04	*R* = −0.06
Oxidative capacity index (s)	10.9 ± 4.7	*R* = 0.12	*R* = 0.28	*R* = −0.02

### Finger flexors' performance

Three tests were used to assess finger flexors' performance. MVC test was completed at the first session to determine the level of finger flexors' strength and to calculate the intensity of the intermittent endurance tests. An all-out test was performed to determine the critical force of the finger flexor as an estimate of metabolic steady and non-steady state delimitation, which was further employed to calculate hanging recovery intensity.

**Figure 2:**
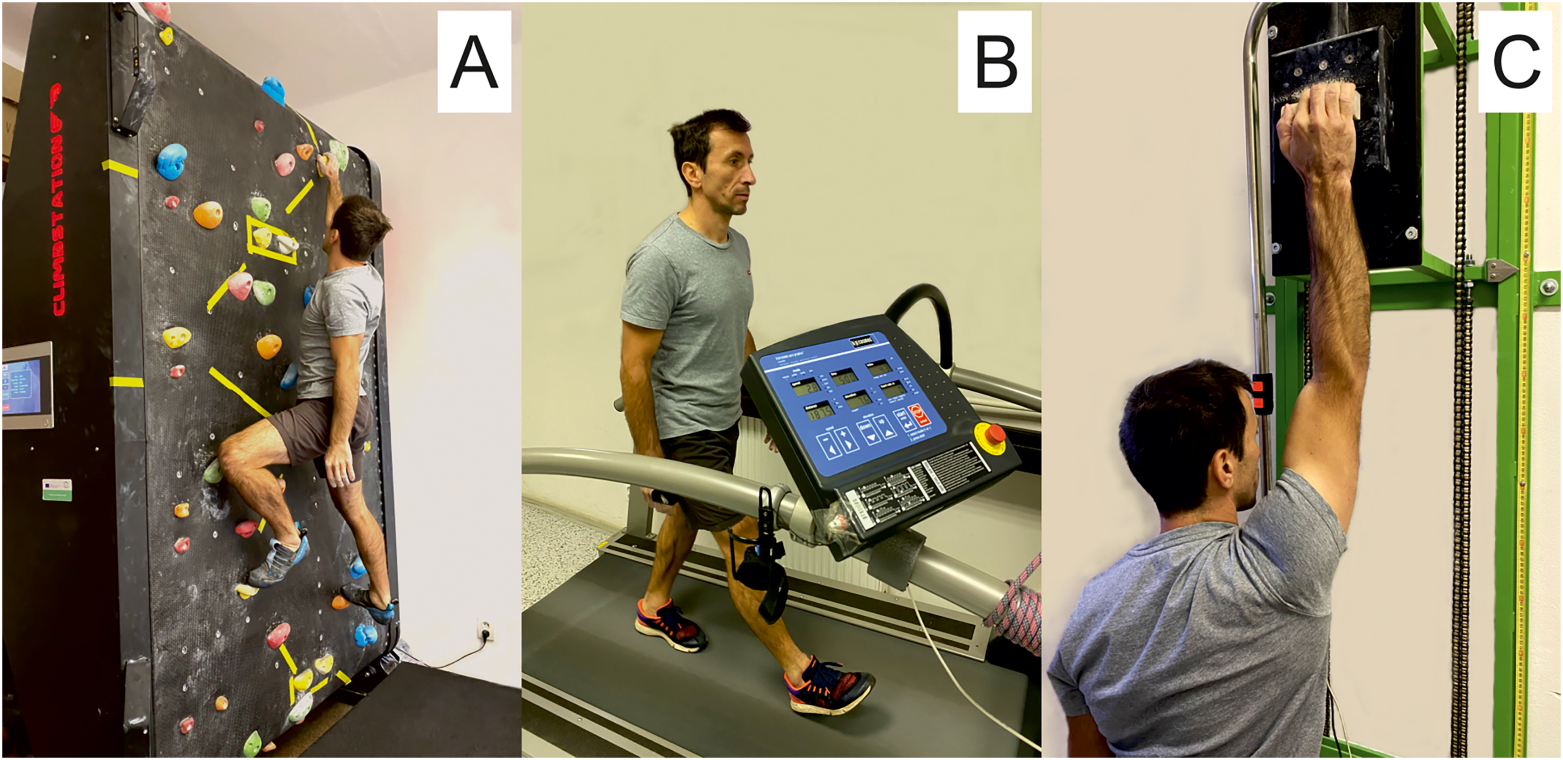
Illustrative photos of active recovery strategies: **(A)** climbing recovery; **(B)** walking recovery; **(C)** hanging recovery with shaking.

All these tests were performed using a climbing-specific handgrip dynamometer (1D—SAC, Spacelab, Sofia, Bulgaria). Specifically, only four fingers without the thumb using an open grip position on the 23-mm-deep wooden hold were applied to maximally activate the flexor digitorum profundus (35). The tests were performed in a standing position as shown in Figure 2C, with the dominant hand and arms in ∼180° shoulder flexion and the elbow slightly flexed (29). During all tests, loud verbal encouragement was provided.

#### MVC test

The participants performed two MVC tests (1 min passive recovery between trials). After an acoustic signal, they progressively pulled on the hold for 5 s to transfer as much of their weight as they could on the hold. If the climber was able to transfer his entire weight to his arm, the weight was gradually increased by placing a kettlebell in the non-dominant arm. The highest value from these two tests represented MVC. The test was shown to be reliable in assessing finger flexors' strength in an ecologically valid setting (29).

#### All-out test

A 4 min all-out test was performed with a work–rest ratio of 7:3 s. During the work phase, the participants were instructed to produce as much force as possible (32). During the relief phase, the participants had their fingers placed relaxed on the hold. To evaluate the critical force, the mean force derived from the last three contractions of the test was defined. The test was shown to be reliable in assessing finger flexor endurance in an ecologically valid setting (36).

#### Intermittent test at 60% MVC

The intermittent handgrip test was used as a performance indicator and further analyzed for AR effectiveness. These tests were performed at the target zone of 60% MVC ± 10% at a work–rest ratio of 8:2 s. During the relief phase, they couldn't shake their forearms or hands. Acoustic and visual feedback for the time and intensity were provided. The test was automatically finished when the force dropped for more than 1 s below the target zone. Time in the target zone and impulse were taken in the analysis. The test was shown to be reliable in assessing finger flexor strength in an ecologically valid setting (29).

#### Incremental treadmill test

HR max was determined using a graded protocol on a treadmill (Quasar, H/P/Cosmos, Germany). The test started at a submaximal speed of 10 km.h^−1^ without any belt inclination (0%) for 4 min. After that, a graded step protocol (+1.5% inclination per minute) was applied until volitional exhaustion.

### Active recovery strategies

Easy climbing (Figure 2A) activating both forearm flexors and lower body muscles, walking (Figure 2B) activating lower body muscles, and hanging (Figure 2C) activating only the dominant forearm flexors were used between exhaustive intermittent tests at 60% in a randomly assigned order, one recovery strategy used per one visit. Each recovery protocol lasted for 22 min and consisted of 3 min of preparation, 3 × 4 min of AR and 2 min of passive recovery in between, and finally 3 min for preparation (Figure 1). During the passive recovery, the participants were asked to stay calm in a sitting position.

#### Climbin*g*

During the climbing recovery, the participants climbed an easy route (∼3 IRCRA) on a 3.8 m high motorized treadwall (ClimbStation generation 1, Forssa, Finland) with a 6-m-long belt. The climbing speed was set to 6 m·min^−1^. The intensity was adjusted by the inclination of the belt from +3 to +9% (positive angle) based on the actual average muscle oxygenation in the forearm (see Muscle tissue oxygenation). The participants were instructed to shake out their hands at each movement.

#### Walking

During walking on the treadmill, a constant speed of 6 km.h^–1^ was applied, and the load was regulated by the inclination of the belt so that the HR corresponded to 60%–65% of the individual HR max. Initially, the belt was set to 5%, and after 30 s, the inclination was adjusted up or down by 1% to achieve the target HR zone. The inclination was recorded in the testing protocol and served as the starting inclination for the subsequent walking recoveries.

#### Hanging

Hanging recovery was performed using only the dominant arm and the same hold and grip position as for finger flexors' tests. However, the contractions were completed at the work–rest ratio of 5:5 s at 50% of the critical force derived from the 4 min all-out test to ensure that the intensity was far below the maximal metabolic steady state. During the relief phase, the participants were allowed to shake their hands near the body (to enhance recovery). The intensity was set based on pilot works which showed that most participants find the load easy to perform and the forearm muscle oxygenation does not drop significantly under the resting values.

### Muscle tissue oxygenation

A continuous wave NIRS device (Portamon, Artinis Medical System, BV, Netherlands) was used to monitor tissue oxygenation on the dominant forearm over the FDP. The measurements were taken throughout all tests and recoveries. The NIRS device was placed according to Fryer et al. (37) using bi-adhesive tape and covered with black tape so that it would hold firmly during testing and no light would pass through. To ensure that the device was placed in the same location, permanent pen markers were used on the skin. Measurements were taken after intermittent tests at 60% MVC, where the participants sat down and placed their hands against their body, and tissue oxygenation values were measured for 1 min.

The tissue oxygenation index (StO_2_) was used as a proxy measure of tissue oxygenation and muscle perfusion (38). From the intermittent tests at 60% MVC, minimum oxygen saturation (StO_2 min_) was analyzed. StO_2 min_ represents the level of muscle oxygen desaturation which was found to be closely associated with muscle endurance performance (1, 39). Additionally, to compensate for different resting StO_2_ values, we used the degree of deoxygenation (ΔStO_2_) which was calculated as differences between StO_2 min_ and resting values. Moreover, from intermittent tests at 60% MVC, the rate of deoxygenation (StO_2_ rate) was calculated as the slope decrease of StO_2_ during the first contraction. The slope of the curve (rate of change) can predict the time to performance failure and can also be an indicator of fatigue because the greater the negative rate, the slower oxygen utilization occurs (40, 41). The recovery tissue oxygenation (StO_2 recovery_) was calculated as the mean StO_2_ during the active phase of recovery. Additionally, we calculated changes in mean tissue oxygenation (ΔStO_2__mean)_ during the recovery phases as a difference between StO_2__recovery_ from the AR and resting values. Values in the positive range indicated higher tissue oxygenation during AR compared to rest, suggesting that enhanced oxygen delivery, rather than utilization, occurred due to increased blood flow, greater capillary bed vasodilation, and/or decreases in the metabolic demands of the muscle.

### Perceived recovery rating

After each recovery strategy, the participants were asked for a subjective perception rating of recovery quality (TQR) on a scale from 6 to 20 (42). Higher scores were associated with a more positive perception of recovery.

### Statistical analysis

Descriptive statistics (mean ± SD) were used to characterize anthropometric and performance variables in all participants. Normality was tested using Shapiro–Wilk tests. The effect of the recovery strategy on localized isometric performance was assessed using the impulse and time changes from the isometric intermittent tests. Moreover, StO_2 min_, ΔStO_2_, StO_2_ rate, and ΔStO_2__mean_ were evaluated, and the differences were assessed using repeated-measure ANOVA (recovery strategy × number of trials) with Bonferroni correction to examine specific pairwise differences. Statistical significance was set to *P* < 0.05. To assess the effect size, partial eta-squared (*η*^2^) was calculated for ANOVA, and paired differences between repetitions and AR strategies were analyzed by Cohen's delta (*d*). All analyses were conducted using the SPSS statistical software (IBM Corp. Released 2019. IBM SPSS Statistics for Windows, Version 26.0. Armonk, NY, USA).

## Results

### Performance

**Figure 3:**
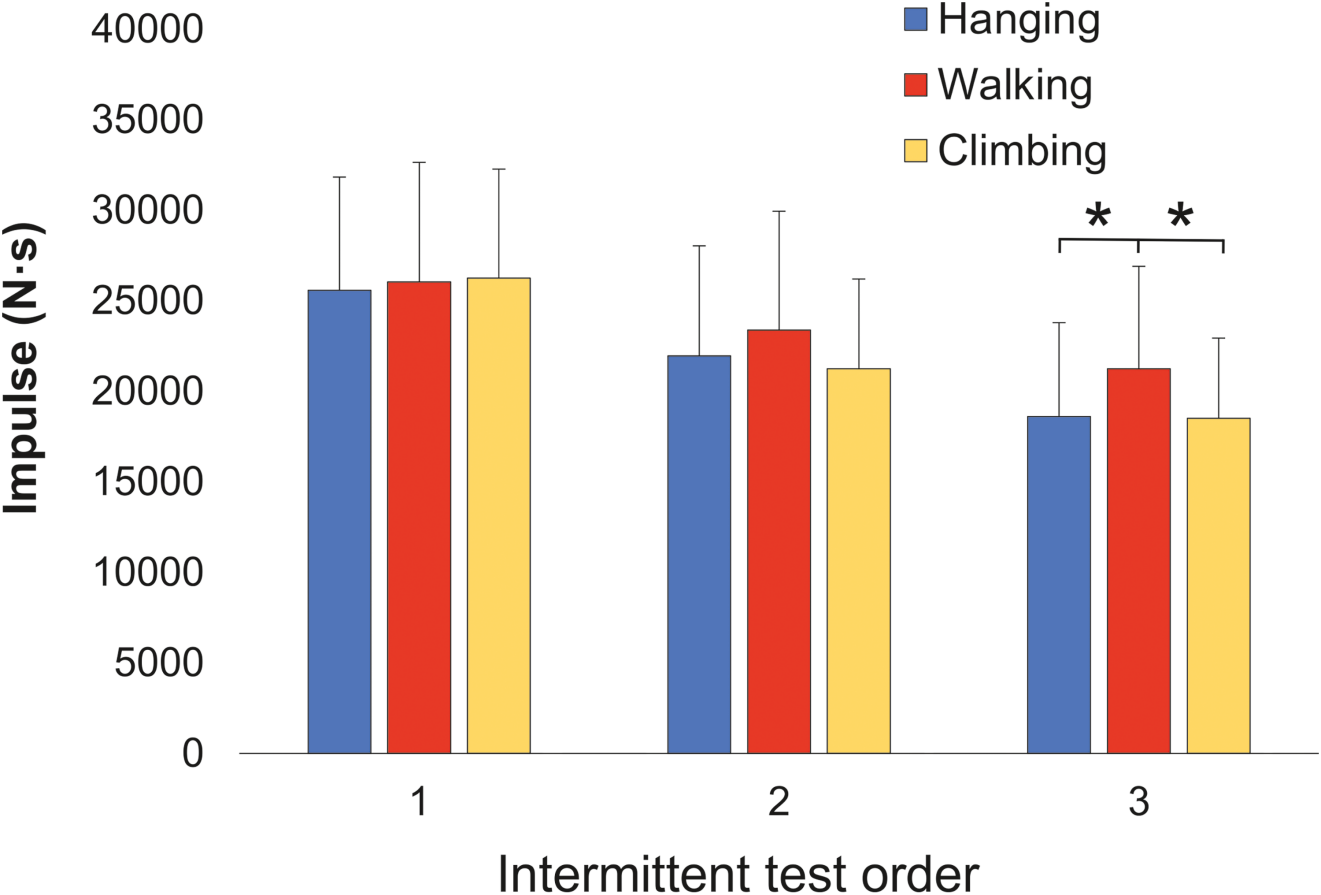
Mean impulse from the three exhaustive intermittent tests for three active recovery strategies: intermittent hanging, walking, and easy climbing. *Significant (*P* < 0.05) differences between walking-hanging and walking-climbing in the third trial.

The mean time (∼76 s, Table 2) and impulse (Figure 3) for the first trials at the intermittent test were the same in all recovery strategies (*P* > 0.05). The impulse significantly decreased from the first to third trials after all AR strategies (main effect of test repetition: *P* < 0.001, *η*^2^ 0.77). Moreover, a significant interaction of AR strategy and trial number was found (*P* = 0.04, *η*^2^ = 0.14).

**Table 2:** Mean (*±*SD) time to exhaustion and tissue oxygenation responses from the intermittent finger flexors' tests. Minimum oxygen saturation (StO_2 min_) represents the lowest tissue oxygenation index during the test. The degree of deoxygenation (ΔStO_2_) was calculated as differences between resting values and StO_2 min_. The rate of deoxygenation (StO_2_ rate) was calculated as a decrease of StO_2_ in time during the first contraction. Evaluation of tissue oxygenation was only possible in 13 participants.

	*N*	Intermittent test order	Hanging	Walking	Climbing
Time (s)	17	1	76.2 ± 17.6^†^	76.6 ± 18.5^†^	76.9 ± 16.7^†^
		2	65.0 ± 16.1^†^	69.3 ± 18.6^†^	63.0 ± 12.5^†^
		3	55.0 ± 14.5*^†^	62.4 ± 13.1*^†^	54.4 ± 10.5*^†^
StO_2 min_	13	1	27.0 ± 9.2	29.7 ± 6.9	26.4 ± 7.1
(%)		2	29.6 ± 8.2	30.4 ± 6.2	28.0 ± 6.5
		3	29.6 ± 7.6	31.3 ± 4.8	27.9 ± 6.4
ΔStO_2_	13	1	25.9 ± 9.5	22.4 ± 8.1	26.8 ± 7.7
(%)		2	23.3 ± 8.9	21.7 ± 6.9	25.2 ± 8.4
		3	23.4 ± 9.1	20.8 ± 5.9*	25.4 ± 7.4*
StO_2_ rate	13	1	−2.0 ± 0.8	−2.1 ± 0.9	−2.0 ± 1.0
(%·s^−1^)		2	−2.6 ± 1.4	−2.2 ± 1.0	−2.0 ± 0.9
		3	−2.6 ± 1.1	−2.5 ± 1.2	−2.0 ± 0.5

* Significant (*P* < 0.05) differences between walking, hanging, and climbing in the third trial.

^†^ Significant (*P* < 0.05) effect of test's trial (repetition).

**Figure 4:**
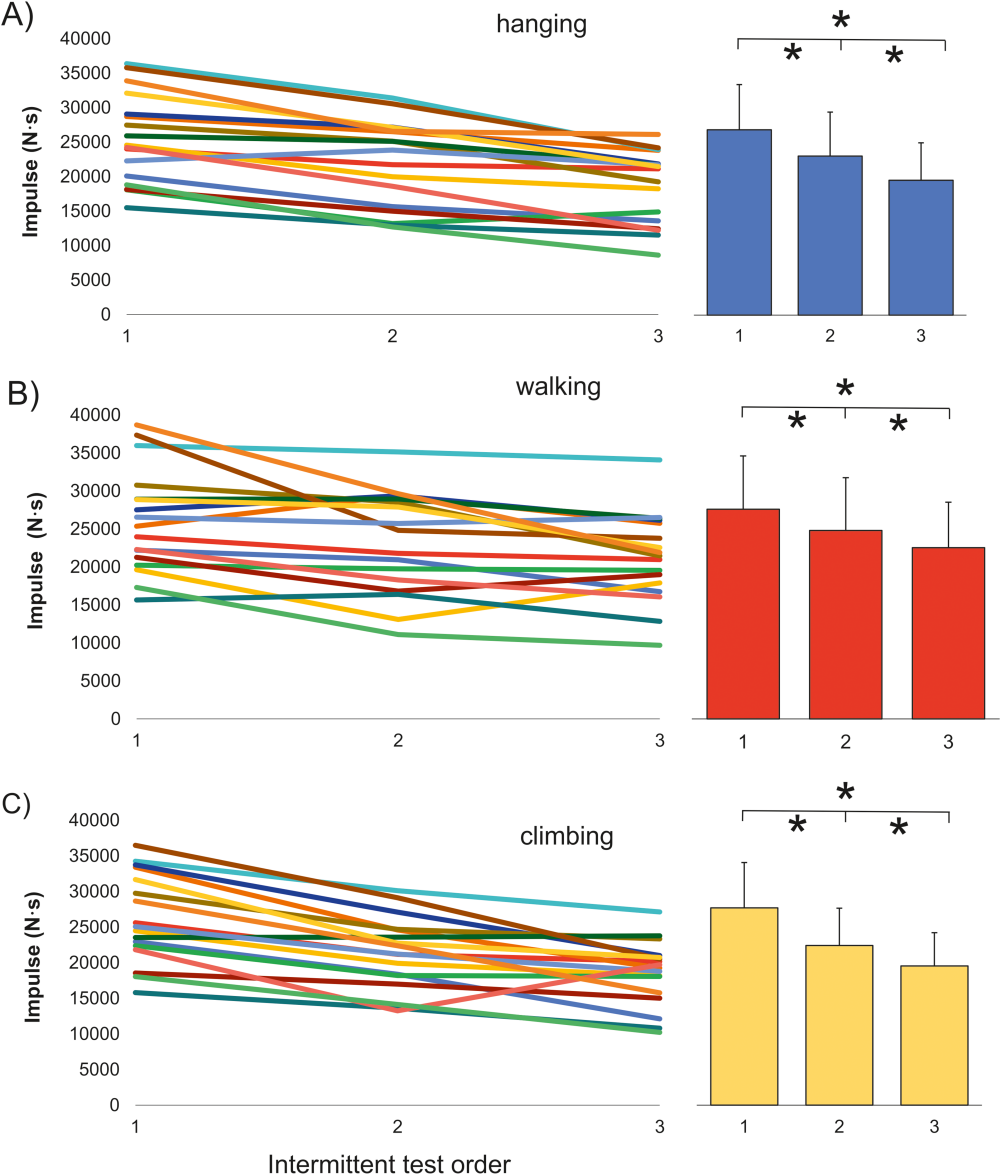
Individual responses and mean impulse from the three exhaustive intermittent tests after different recovery methods. **(A)** Hanging; **(B)** walking; **(C)** climbing. *Significant (*P* < 0.05) differences between trials after the active recovery method.

Pairwise comparisons demonstrated performance decrease from the first to third trials after walking (−18.4%, *P* = 0.002, *d* = 0.78), climbing (−29.5%, *P* < 0.001, *d* = 1.48), and hanging (−27.2%, *P* < 0.001, *d* = 1.22) (Figure 4). There were no significant (*P* > 0.05) differences between recovery strategies in the second trial. However, in the third trial, the impulse from the intermittent test after walking (21,253.8 ± 5,650.1 Nꞏs) was significantly higher than after hanging (18,618 ± 5,174 Nꞏs, *P* = 0.013, *d* = 0.49) and after climbing (18,508 ± 4,435 Nꞏs, *P* = 0.009, *d* = 0.54) (Figure 3). The time changes after AR and their significance mirrored those of impulse (Table 2). No relationship was observed between performance decrease and training or climbing ability characteristics or oxidative capacity index (Table 1).

The ΔStO_2_ was significantly higher (*P* = 0.043, *η*^2^ = 0.23) after climbing than walking during the third trial (Table 2). No significant interactions (*P* < 0.05) between recovery strategy and trial number for ΔStO_2_, StO_2 min_, and StO_2_ rate were found.

### Recovery

There were no significant (*P* > 0.05) differences in the ΔStO_2 recovery_ and StO_2 recovery_ between recovery phases of the same AR. However, ΔStO_2 recovery_ was significantly higher in hanging than in climbing at the first (*P* = 0.007, *d* = 0.93) and second (*P* = 0.033, *d* = 0.71) recovery phase. Similarly, ΔStO_2 recovery_ was significantly higher in walking than in climbing, but only at the first phase (*P* = 0.027, *d* = 1.38). Moreover, StO_2 recovery_ was significantly higher in hanging than in climbing at the first (*P* = 0.005, *d* = 1.32) and second (*P* = 0.025, *d* = 1.56) recovery phase, and StO_2 recovery_ was significantly higher in walking than in climbing, at the first (*P* = 0.003, *d* = 1.58) and second (*P* = 0.002, *d* = 1.52) phases (Table 3).

**Table 3:** Mean ± SD tissue oxygenation responses and perceived recovery quality (TQR) from the three recovery strategies (hanging, walking, and climbing). Each recovery strategy comprised two phases between exhaustive intermittent tests. The changes in mean tissue oxygenation (ΔStO_2 mean_) during the recovery phase were calculated as a difference between resting values and StO_2 recovery_. The recovery tissue oxygenation (StO_2 recovery_) was calculated as mean StO_2_ during AR. Evaluation of tissue oxygenation was only possible in 13 participants.

	*N*	Recovery phase	Hanging	Walking	Climbing
ΔStO_2 recovery_	13	1	2.8 ± 5.5*	4.6 ± 4.1^†^	−2.9 ± 6.9*^†^
(%)		2	2.9 ± 5.8*	5.3 ± 5.3	−1.9 ± 7.6*
StO_2 recovery_	13	1	55.7 ± 3.7*	56.7 ± 3.6^†^	50.3 ± 4.8*^†^
(%)		2	55.8 ± 3.1*	57.4 ± 4.1^†^	51.4 ± 4.2*^†^
TQR	17	1	13.7 ± 2.4	15.0 ± 2.2	15.1 ± 1.5^‡^
		2	13.5 ± 2.1	14.5 ± 1.8	14.5 ± 1.8^‡^

* Significant (*P* < 0.05) differences between climbing and hanging at a similar repeated level.

^†^ Significant (*P* < 0.05) differences between climbing and walking at a similar repeated level.

^‡^ Significant (*P* < 0.05) effect of test's phase.

TQR score after intermittent hanging was non-significantly (*P* = 0.06–0.10, *d* = ∼0.5–0.8) lower than after climbing or walking. TQR was significantly (*P* = 0.044, d = 0.40) higher in the first than in the second phase for climbing recovery, and there were no significant differences (*P* > 0.05) for other recovery forms (Table 3).

## Discussion

The study aimed to evaluate the impact of different active recovery strategies on repeated finger flexor intermittent performance leading to exhaustion. Easy climbing or isolated forearm contractions emerged as the least efficient AR strategy to maintain subsequent performance in comparison to walking, indicating that using the same muscle group for AR should be avoided between repeated exhaustive isometric contractions. We observed a significant 18%–30% decrease in performance between the first and third trial after all forms of AR indicating that a 22 min AR period is insufficient for full recovery in the population of male intermediate to elite climbers.

Our results suggest that employing small muscles extensively used in the exhaustive trial is the least efficient AR strategy following localized exhaustive contractions. Fujita et al. (20) argued that 20 min of leg or arm cycling AR is more advantageous than passive recovery between 40 s all-out cycling exercises. Moreover, leg AR led to higher total work in the second trial than arm AR due to improved oxidative functions suggesting that the same muscle groups should be used for AR as for the exhaustive exercise, which is in contrast to our findings. The contradictions may likely be attributed to the amount of muscle groups involved in AR determining the systemic cardiovascular responses and metabolites cleared. Easy AR contractions of forearm muscles cannot provide such a metabolite clearance capacity as large muscle groups are involved when walking.

Oxidative functions during exercise and recovery have been assessed using StO_2_ dynamics. The StO_2_ reflects the dynamic balance between muscle O_2_ supply and demand, where high values represent high muscle perfusion and low metabolic demands, while low values are associated with limited blood flow and/or high O_2_ consumption (38). The magnitude and rate of StO_2_ decrease are correlated to higher VO_2__max_, critical power, improved oxidative functions, or localized muscle performance (1, 43–45). Based on our NIRS data, neither the magnitude nor the rate of desaturation showed changes in localized oxygen dynamics during exhaustive finger flexor contractions. Therefore, we cannot confirm any effects of our AR strategies on muscle oxidative functions. The likely explanation for hanging and climbing as the least efficient AR method may be the substrate availability in the repeated performance bouts. It was shown that 15–30 min of AR may mitigate glycogen and PCr resynthesis in the active muscles (46, 47). It was speculated that AR may result in a competition for O_2_ between PCr resynthesis, lactate oxidation, and the increased O_2_ cost of the additional exercise (48). From this perspective, involving large not-fatigued muscle groups for AR may contribute to faster metabolite removal and acidosis decrease on one hand and to spare energy substrates in the fatigued muscle on the other hand. In the current study, we saw higher StO_2 recovery_ during walking and hanging than during climbing. As StO_2_ represents the balance between O_2_ delivery and uptake, a lower StO_2_ in climbing conditions may correspond to greater O_2_ utilization in the forearm muscles. However, the overall level of resting StO_2 mean_ in all AR conditions was high and the increased localized cost of O_2_ using NIRS cannot be confirmed from our data as the blood flow was not measured. Hence, the exact physiological mechanisms of recovery after exhaustive localized contractions are still unknown.

From a psychological perspective, participants' perception of recovery did not align with subsequent performance decreases. Although not significant (*P* = 0.06–0.10), effect sizes (*d* = ∼0.5–0.8) showed that the participants felt less recovered after intermittent hanging than after climbing or walking. Recovery after walking and climbing was perceived similarly. However, the smallest performance decrease was found after walking. This disagrees with the StO_2 recovery_, which was significantly lower during climbing conditions in comparison to walking and hanging. Therefore, it appears that neither TQR nor resting StO_2_ can satisfactorily predict the degree of recovery before subsequent exhaustive performance.

In a climbing-specific setting, Valenzuela et al. (18) explored easy climbing and walking as two distinct AR strategies, concluding that 2 min of easy climbing was more beneficial than 2 min of walking between climbing routes. However, our findings, demonstrating the lowest decrease in repeated performance after walking, contradict these results. This discrepancy may be attributed to variations in AR protocol duration and performance outcomes, making direct comparisons challenging. For example, Valenzuela et al. (18) employed a non-exhaustive climbing protocol, with the speed of ascent as the outcome variable, rather than considering the difficulty of the climb. While the climbing speed affects systemic physiological responses, localized fatigue may be more associated with the climb's difficulty (49). Additionally, only 2 min of active recovery may have compromised the efficiency of AR, as longer periods have been shown to be more beneficial (11). Therefore, we believe that our proposed experimental design may better reflect real climbing-specific conditions while maintaining high internal validity.

Studies on AR in sports requiring finger flexor strength have typically examined a single AR strategy in comparison to passive or alternative recovery methods. The execution of AR involved 20 min of 30–40 W cycling (13), 10 min of 25 W cycling (23), or 3.5 min of moderate and fast walking (12). Despite a wide variety of AR approaches, all studies reported a superior effect of AR over passive recovery. The significant decrease in finger flexor muscle performance in the current study for all AR forms was unexpected in this population of climbers accustomed to repeated exhaustive climbs. Using a similar study design and finger flexor muscle performance, Kodejška et al. (50) found a 22% impulse decrease between the first and the third test after passive recovery, a roughly similar decrease to our study. We did not include passive recovery in our study, and therefore, we can only speculate if our AR strategies would be more efficient than passive recovery. However, the superior efficacy of AR over passive recovery is well-documented in repeated bouts of performance relying highly on glycolysis (13, 19). Similar to our study, Valenzuela et al. (18) found a significant climbing performance decrease between the first and third trials but separated by a very short 2 min of AR. With a longer AR duration, no changes in repeated performance were found for exhaustive climbing (13). The discrepancies in recovery speed may be due to the selected population (lead climbers vs. boulders, experience, climbing ability, type of training) or different ambient temperatures (51, 52). The absence of a clear relationship between performance decrease and various factors, such as muscle oxidative capacity, climbing ability, experience, or training characteristics, suggests that more complex factors may influence the recovery process, possibly necessitating a larger sample size and additional physiological markers for meaningful analysis.

While isolated local contractions offer insight into physiological responses, their direct transferability to complex sport performance in climbing is not straightforward, as it depends on various mechanisms. Our study specifically focused on male intermediate to elite climbers, limiting the generalizability of results to the broader population or different experimental settings. Other limitations of the study are the incomplete NIRS data as four records could not have been used due to technical issues. Therefore, a slight bias in the results may have occurred.

## Conclusion

The results of this study highlight the deteriorating effect of climbing or intermittent hanging as an AR strategy compared to walking between exhaustive finger flexor intermittent contractions. Our findings suggest that utilizing large muscle groups not directly involved in the exercise is a more effective approach for AR than targeting the muscles directly engaged in the exhaustive contractions. However, it is noteworthy that even with a 22 min AR period, complete recovery may not be achieved following exhaustive contractions lasting approximately 1.0–1.5 min. Coaches and athletes should take this into consideration when planning recovery periods or exploring alternative strategies. Additionally, the limited validity of perceived recovery highlights the need for a more nuanced and evidence-based approach to recovery assessment in the context of sport climbing and similar activities.

## Data Availability

The original contributions presented in the study are included in the article/Supplementary Material, further inquiries can be directed to the corresponding author.
